# Vacuum-Assisted Needle-Free Capillary Blood Sampling

**DOI:** 10.1177/19322968231161361

**Published:** 2023-03-17

**Authors:** Michael Hoffman, James McKeage, Bryan Ruddy, Poul Nielsen, Andrew Taberner

**Affiliations:** 1 Auckland Bioengineering Institute, The University of Auckland, Auckland, New Zealand; 2 Department of Engineering Science, The University of Auckland, Auckland, New Zealand

**Keywords:** needle-free, jet injection, glucose, diabetes, blood sampling, capillary blood

## Abstract

**Background:**

Poor glycemic management persists among people practicing insulin therapy in relation to type 1 and 2 diabetes despite a clear relationship with negative health outcomes. Skin penetration by jet injection has recently been shown as a viable method for inducing blood release from fingertips. This study examines the use of vacuum to enhance the volume of blood released and quantifies any dilution of the collected blood.

**Methods:**

A single-blind crossover study involving 15 participants, each receiving four different interventions, was conducted wherein each participant served as their own control. Each participant experienced fingertip lancing and fingertip jet injection, both with and without applied vacuum. Participants were divided into three equal groups to explore different vacuum pressures.

**Results:**

This study found that glucose concentration in blood collected under vacuum following jet injection and lancing were equivalent. We found that applying a 40 kPa vacuum following jet injection produced a 35-fold increase in the collected volume. We determined the limited extent to which the injectate dilutes blood collected following jet injection. The mean dilution of blood collected by jet injection was 5.5%. We show that jet injection is as acceptable to patients as lancing, while being equally suited for conducting glucose measurements.

**Conclusions:**

Vacuum significantly enhances the volume of capillary blood released from the fingertip without any difference in pain. The blood collected by jet injection with vacuum is equivalent to that from lancing for glucose measurement purposes.

## Introduction

People with type 1 and insulin-dependent type 2 diabetes require injections of insulin multiple times per day to manage the glucose concentration in their blood. Frequent administration of insulin has been demonstrated to improve patient outcomes.^1-3^ The appropriate dose of insulin is determined using a measurement of blood glucose concentration, which typically requires the fingertip to be pricked with a lancet. The drop(s) of blood resulting from this prick is used to perform the glucose measurement. In an effort to make this process easier for people with diabetes, we are investigating whether glucose testing can be integrated into a needle-free jet injection device, thus totally avoiding the need for a lancet to prick the skin. Performing this process without a needle/lancet will avoid the associated issues of sharps waste, accidental needle sticks, and needle phobia.^4-6^

Jet injection can release capillary blood for glucose testing by using a high-speed jet of liquid, in place of the lancet, to break the skin. A recent study investigating the relationship between the shape of the jet during injection and the volume of blood released demonstrated that sufficient blood for a glucose test was released by jet injection, although the jet injection released less blood than a lancet.^7^ However, it is essential to understand the extent to which the sample is diluted by any injectate residue if a glucose concentration measurement is to be conducted upon the sample. To measure the fraction of injectate in the blood collected (the dilution), a laser fluorimeter has been developed.^8^

The application of vacuum following lancing has been shown to increase the volume of blood released.^9^ This study examines the application of vacuum after jet injection to significantly increase the volume of blood released. In this study, human participants received four interventions of both lancing and jet injection of fingertips, with and without vacuum. Measurements of blood volume, glucose concentration, and dilution were collected, along with self-reported pain and acceptability scores associated with each intervention. By enhancing the volume of blood released in this way, it will be possible to collect a sample with reduced discomfort to the participant.

## Methods

### Participant Recruitment

This study was approved by the Northern B Health and Disability Ethics Committee of New Zealand (2021 FULL 11035, registration: ACTRN12621001572853, www.anzctr.org.au). This study was advertised within the Auckland Bioengineering Institute at the University of Auckland. The trial involved a single visit followed by a questionnaire the next day. Each participant provided a written declaration of consent after being briefed by the research nurse on site.

Fifteen participants aged between 24 and 52 were recruited for the study. Key exclusion criteria included insulin-dependent diabetes, hemophilia (or other bleeding/clotting disorders), being a carrier of any blood-borne infectious agent (eg, human immunodeficiency virus [HIV] and hepatitis B virus [HBV]), reduced peripheral circulation (eg, from Raynaud’s disease or beta blocker use), amputation affecting a number of fingertips, and allergy to iodides and/or indocyanine green (ICG).

### Study Design

#### Interventions

Each participant was subjected to four capillary blood sampling interventions. These interventions were performed on the side of the fingertip of the middle (third finger) and ring finger (fourth finger) of either hand. Two of the four interventions used a lancet (ACCU-CHEK Safe-T-Pro Plus, Roche, Basel, Switzerland) to prick the finger to release the capillary blood sample. This is the current best practice recommended by the World Health Organization.^10^ Vacuum was applied to the fingertip following one of the lancet pricks.

The remaining two interventions used a needle-free jet injection in place of the lancet to break the skin.^7^ This device was driven by an electric motor with a portable power amplifier and controller.^11^ This injector used a thin stream of fluid (<0.05 mL) to break the skin, targeting the same penetration depth as a lancet (2.3 mm). The fluid used in the injector was an isotonic saline solution marked with a low concentration (10 mg/L) of ICG (Verdye, Diagnostic Green). Indocyanine green is a water-soluble fluorescent dye that was originally approved by the Food and Drug Administration for intravenous diagnostic applications such as angiography at a concentration of 5 g/L. A working ICG concentration of 10 mg/L was prepared from a stock 5 g/L with buffered isotonic saline.

Subsequent to the intervention, the blood released was collected into a glass capillary tube (0.2 mm by 4 mm by 40 mm, VitroCom, NJ) at 20-second intervals, a total of three times. Vacuum was reapplied following the first and second collection such that three sequential samples were collected following a 10-second application of vacuum. Where vacuum was not applied, the operator applied a gentle pressure to the fingertip between collection intervals.

The study was divided into three arms that differed only in the magnitude of the vacuum pressure applied following two of the four interventions. All participants received the same two lancet pricks and two jet injections, with one jet injection and one lancet prick followed by the application of vacuum. Each participant thus served as their own control. Participants were randomly assigned to one of the arms of the study as follows:

Arm 1: −20 kPa gauge pressure applied following 2/4 interventions
Arm 2: −40 kPa gauge pressure applied following 2/4 interventions
Arm 3: −60 kPa gauge pressure applied following 2/4 interventions

All participants received all four interventions with the order and location (which fingertip) of each intervention randomized. The interventions were performed at intervals of approximately five minutes with a research nurse present. The participants were blinded by an opaque barrier that prevented them from observing the procedure.

#### Vacuum system

A diaphragm pump (D2028B, Airpo) was used to generate a controlled vacuum pressure using a custom system controlled by a LabVIEW (National Instruments, 2022) virtual interface. An absolute pressure sensor (NXP Semiconductors, MPX5700) was incorporated to provide real-time feedback on the pressure achieved. The pressure was sampled at 40 kHz with a NI-9263 module (National Instruments) and fed into a proportional–integral–derivative (PID) controller to operate the pump. The operator selected from −20, −40, and −60 kPa as the target set point pressure for the system. Testing the system against a reference pressure sensor (DP-001-P, Panasonic Industry Co., Ltd) showed that the system was able to maintain pressure within 1 kPa of the target value. The prescribed pressure was then delivered via connective tubing to the terminating suction cup that had an internal diameter of 8 mm. The LabVIEW control software limited the time of applied vacuum to 10 seconds.

#### Fluorimeter

A fluorimeter was adapted from Madadkhahsalmassi et al^8^ to infer the concentration of the fluorescent marker (ICG) in the resulting blood samples, and thus provided a measurement of the extent to which the blood sample was diluted with the injectate used to break the skin. A 780-nm laser (US-lasers D7805I) was used to excite the ICG. The fluorescent emission from the incident laser was measured using a photomultiplier tube (Hamamatsu H7422). Samples were retained in glass capillary tubes (0.2 mm by 4 mm by 50 mm, #3524 Vitrocom) before being further examined.

### Outcome Variables

#### Blood dilution

After each collection, the capillary tube containing extracted blood was inserted into the laser fluorimeter to measure dilution. The samples were manually aligned before triggering the fluorimeter. The fluorescent responsivity and transmission were recorded. Dilution was calculated from a calibration curve prepared by making known dilutions from porcine blood as previously established.^8^

#### Blood volume

After each collection, the capillary tube containing extracted blood was imaged under a digital microscope (Digitech QC3199, Digitech industries, Sheung Wan, Hong Kong) with a transmission light source. The sample volume was calculated from the pixel area given the known internal dimensions of the capillary tubes. Images were processed with automated segmentation in Python (v2.7.9) using the OpenCV-Python library (v4.6.0). Measurements performed on known volumes indicated that this method provided an root mean square (RMS) error of 2.6%.

#### Pain and acceptability

After each intervention and blood collection, the participant was asked to assign a pain and acceptability a score. For pain, a score from 0 to 10, with 0 representing “no pain” and 10 representing “extremely severe pain.” For acceptability, a score from 0 to 10, with 0 representing “completely unacceptable” and 10 representing “extremely acceptable.”

A digital microscope was used to image the intervention site immediately following the completion of all interventions to record any swelling or bruising. The participants were asked to complete a questionnaire 24 hours later to assess residual pain and discomfort.

#### Glucose concentration

Following the microscope imaging of each collection tube, if sufficient volume was collected, the blood was dispensed into single-use glucose test strip. The glucose concentration of each sample was measured using a glucometer (CareSens N Premier; i-SENS, Inc, Seoul, Korea). This device has a rated accuracy of ±15.0 mg/dL for glucose concentration <100 mg/dL.

### Statistical Analysis

Statistical power calculations were made with a type I error rate (*a*) of 0.05 and a type II error rate (ß) of 0.2 for group sizes of five participants to enable the establishment of significant differences in the mean volume released as low as 1.4 µL (σ_*BloodVolume*_ = 0.7 µL). These calculations showed that differences in mean blood dilution as low as 2% between groups (σ_*fluorimeter*_ = 1%) can be determined, and differences between groups whose mean glucose concentration differs by as little as 10% (σ_*glucometer*_= 5 mg/dL) can be determined.

The data collected across the four interventions were analyzed using the statsmodels (v0.9.0) and SALib (v1.4.5) libraries in Python (v2.7.9). The pairwise_tukeyhsd method was used to perform a Tukey’s range test to compare each measured outcome. An equivalence test was performed using the ssw.ttost_ind method to identify a clinically significant difference in glucose concentration between paired measurements following jet injection with vacuum and lancing with vacuum. Due to the limited number of samples collected following jet injection alone, some comparisons were not possible (eg, sample dilution for jet injection samples without suction).

## Results

Sixteen participants were recruited in total; one participant was withdrawn from the study. The relevant demography is summarized in Table 1.

**Table 1.** Demographic Characteristics of Participants Recruited.

	Recruitment (n = 16)	Completed (n = 15)
Age (years; mean [SD])	31.0 (7.6)	31.5 (7.6)
Sex (female; n [%])	3 (18.8%)	3 (20%)
Sex (male; n [%])	13 (81.2%)	12 (80%)
Dominant hand (Left; n [%])	2 (12.5%)	2 (13.3%)
Dominant hand (Right; n [%])	14 (87.5%)	13 (87.5%)

### Blood Volume and Concentration

**Figure 1.**
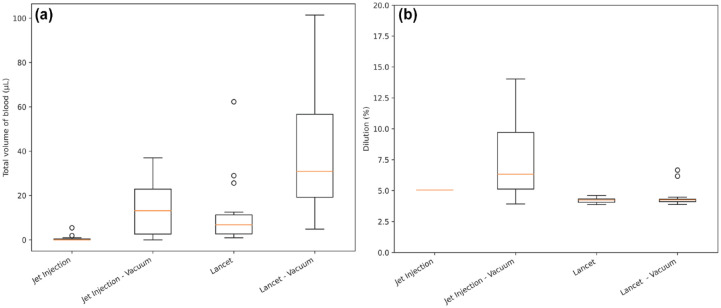
(a) (Left)—Total volume across the three collection points associated with each intervention. (b) (Right)—Dilution of each collected sample associated with each intervention.

Blood volumes collected under vacuum (Figure 1a) showed an appreciable increase over those collected without vacuum. Blood volumes collected by lancing were consistently greater than those from a jet injection. The measured dilution of samples collected by lancing was consistent across applied vacuum pressures (Figure 1b).

There was a consistent increase in volumes collected with vacuum pressure applied from 0 to 40 kPa for both methods (Table 2). The volume of blood collected under a vacuum pressure of 60 kPa was less than the volume collected under 40 kPa.

**Table 2.** Data Values for Volume and Dilution Measurements Taken for Each Intervention Associated by Vacuum Pressure.

		Previous study^7^	Vacuum pressure			
		No vacuum (mean [SD])	0 kPa (mean [SD])	20 kPa (mean [SD])	40 kPa (mean [SD])	60 kPa (mean [SD])
Total intervention volume (µL)	Lancet	4.5 (3.4)	12.0 (15.8) *^†^	34.0 (14.1) *^†^	57.3 (32.0) *^†^	22.9 (12.7) *^†^
	Jet injection	0.8 (0.7)	0.6 (1.4) *^†^	10.0 (6.9) *^†^	22.0 (11.5) *^†^	16.2 (16.2) *^†^
Sample dilution (%)	Lancet	9.3 (4.5)	1.3 (1.2)	1.0 (0.6) ^†^	1.5 (1.4) ^†^	1.3 (0.7) ^†^
	Jet injection	18.5 (11.2)	N/A	5.7 (4.9) ^†^	6.0 (4.3) ^†^	4.2 (2.7) ^†^

* Comparison across vacuum pressure *P* < .05.

† Comparison across intervention method *P* < .05.

Over time (Table 3), the fluid samples collected by jet injection showed a low level of dilution that diminished by the third sample. The lancet samples showed the same dilution as a lancet with vacuum. Dilution was not reliably measured in sufficient numbers of jet-injection-without-vacuum samples due to the low collection volume. The minimum volume required for reliable measurement of dilution was 3 µL. There was no significant difference between the volume of blood collected at each point in the collection sequence.

**Table 3.** Data Values for Volume and Dilution Measurements Taken From Each Blood Sample Collected in Association With the Given Intervention.

		Collection sequence		
		First (mean [SD])	Second (mean [SD])	Third (mean [SD])
Collection volume (µL)	Lancet	2.91 (3.99) ^†^	5.14 (6.66) ^†^	3.96 (5.72) ^†^
	Jet injection	0.18 (0.41) ^†^	0.44 (1.10) ^†^	0 (0.01) ^†^
	Lancet with vacuum	9.84 (9.37) ^†^	11.01 (10.79) ^†^	9.24 (8.53) ^†^
	Jet injection with vacuum	5.75 (3.89) ^†^	5.56 (5.58) ^†^	4.33 (6.19) ^†^
Sample dilution (%)	Lancet	1.69 (0.79) ^†^	1.27 (1.11) ^†^	1.28 (1.27)
	Jet injection	N/A	N/A	N/A
	Lancet with vacuum	1.49 (1.28) ^†^	1.30 (1.02) ^†^	0.91 (0.62) ^†^
	Jet injection with vacuum	6.84 (3.76) *^†^	5.87 (4.71) *^†^	2.12 (1.60) *^†^

Jet injection sample volumes were too low for a fluorescence measurement to be made, prohibiting a dilution measurement.

* Comparison across collection sequence *P* < .05.

† Comparison across Intervention method *P* < .05.

### Blood glucose

The mean glucose concentration measured following lancing (88.9 ± 10.3 mg/dL) is similar to that from jet injection (92.3 ± 8.5 mg/dL). The test of equivalence resulted in the rejection of the null hypothesis that the difference in the mean glucose values is greater than 15 mg/dL (n = 8, *P* = .019).

### Intervention Pain and Acceptability

There was no difference in pain seen across the different methods of sampling (Table 4). The current standard method for capillary sampling, lancing, scored the lowest average acceptability score. There was no significant difference in pain or acceptability associated with an intervention by the application of vacuum. No participant recorded any amount of lingering pain for any intervention on the following day.

**Table 4.** Data Values for Self-Reported Pain (Lower is Better) and Acceptability (Higher is Better) Measurements Associated by Intervention.

	Lancet (mean [SD])	Lancet with vacuum (mean [SD])	Jet injection (mean [SD])	Jet injection with vacuum (mean [SD])
Perceived intervention pain	3.1 (1.4)	2.9 (1.2)	2.9 (1.8)	3.3 (1.7)
Acceptability of intervention	8.54 (2.00)	8.62 (1.73)	9.10 (1.21)	8.77 (1.70)
Pain after 24 hours	0 (0)	0 (0)	0 (0)	0 (0)

## Discussion

The finding that applying vacuum increases the volume of blood released is consistent with previous studies. Previous explorations found that both the volume and rate of blood release increase with the vacuum pressure applied.^9,12^ It is interesting to note that when the highest level of vacuum was applied in this study (60 kPa), less blood was released than under moderate vacuum (40 kPa) (Table 2). This is possibly due to the occlusion of underlying blood vessels as the suction cup presses down with sufficient force.

The volumes of blood collected by vacuum were much more than the volume required for a glucose test strip. The blood collected by jet injection showed a consistently low level of dilution that diminished over time following the intervention. These results indicate that samples collected by jet injection can be used for test assays without concern for specimen dilution. This is further evidenced by the glucose measurement results, which showed the equivalence between the two approaches. The analysis of glucose measurements was limited to the data from participants where a glucose measurement was successfully obtained by both jet injection and lancing. Benchtop verification showed that glucose measurements were independent of ICG concentration. The samples collected by lancing also showed apparent dilution despite the complete absence of any injectate. This is likely due to a small amount of autofluorescence in the blood and is consistent with autofluorescence observed in porcine blood.^8^

Given that the application of vacuum greatly enhances the volume of blood collected, it may be worthwhile exploring less disruptive jet injection regimes. Previous work showed that a small slot-shaped jet could penetrate the fingertip with less pain elicited.^7^ However, this slot-shaped jet, when used without vacuum, did not reliably release sufficient blood for collection and glucose measurement. By using vacuum to enhance the volume of blood release, jet injection through such a nozzle may offer a less painful alternative to lancing. In addition, vacuum may allow sufficient blood volume to be collected from alternative sites as a less painful option for capillary blood sampling.^13^

**Figure 2.**
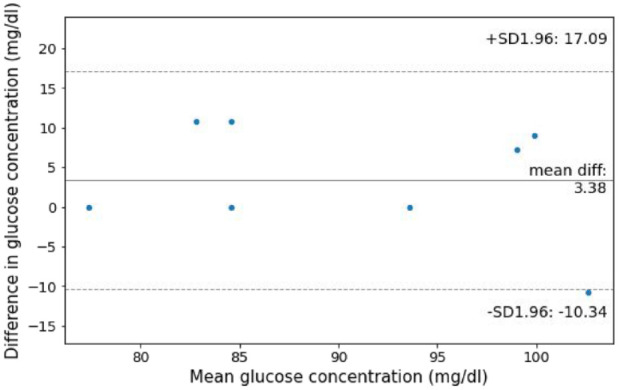
Mean difference plot of glucose measurement pairs in participants with a glucose concentration measurement of both jet injection and lancing samples.

This work highlights the potential to expand the use case of jet injectors to provide needle-free blood sampling in addition to needle-free insulin delivery. The use of controllable, reversible motor driven jet injections offers the ability to both produce the jet to break the skin and vacuum to collect the blood sample.^14^ Jet injection has been used to deliver a range of pharmacological agents, from vaccines to therapeutics.^15^ Jet delivery of insulin is well accepted by people with diabetes as an alternative to needle injection.^16^ In future, a jet-injection device may be able to perform both glucose measurement and insulin delivery as an all-in-one device. This may have the added benefit of reducing the risk of blood-borne virus transmission associated with needle use.^17^ Future work was needed to develop a jet injection system capable of generating a vacuum and delivering a subsequent injection of insulin. Extensive testing is required to ensure that the use of jet injection for blood sampling reduce the accuracy of glucometer measurements below regulatory requirements. Although some injectate remains in blood samples collected by jet injection, it is conceivable the variance of glucose measurements may be reduced by the increased volume of blood collected. The results shown in Figure 2 are suggestive that jet injection does not significantly impact the glucose concentration measured.

## Conclusions

Applying vacuum significantly enhances the volume of capillary blood released from the fingertip following lancing or needle-free injection. This study is the first to demonstrate that there is no statistical difference between glucose concentration in blood collected under vacuum following jet injection and lancing. We are also able to determine the limited extent to which the injectate dilutes blood collected following jet injection. We show that, when used in combination with vacuum, jet injection is as acceptable as lancing without additional pain, while being equally suited for glucose measurements. These findings highlight the potential for blood sampling by smaller penetrations, whether lancet or jet injection, to reduce the pain elicited during the process.
